# Obesity of Sows at Late Pregnancy Aggravates Metabolic Disorder of Perinatal Sows and Affects Performance and Intestinal Health of Piglets

**DOI:** 10.3390/ani10010049

**Published:** 2019-12-25

**Authors:** Chuanshang Cheng, Xiaoyu Wu, Xiaofeng Zhang, Xiu Zhang, Jian Peng

**Affiliations:** 1 Department of Animal Nutrition and Feed Science, College of Animal Science and Technology, Huazhong Agricultural University, Wuhan 430070, China; chengcs1989@gmail.com (C.C.); 13586414898@163.com (X.W.); zhangxiu199205@163.com (X.Z.); 2 Key Laboratory of Animal Nutrition and Feed Science, Ministry of Agriculture and Rural Affairs, WENS Research Institute (Technology Center), Yunfu 527300, China; 3 College of Life Sciences, Zhaoqing University, Zhaoqing 526061, China; xiaofengzhang0413@126.com; 4 The Cooperative Innovation Centre for Sustainable Pig Production, Wuhan 430070, China

**Keywords:** sow, metabolic syndrome, obesity, intestinal health, piglet performance

## Abstract

**Simple Summary:**

Our novel findings suggest that excessive backfat thickness of sows at days 109 of gestation exacerbates the metabolic disorder of perinatal sows, reduces the number and litter weight of piglets born alive, and adversely affects the intestinal health of sows and their offspring piglets. Moreover, the current study also provides an important theoretical reference for strengthening the control of body condition in sows during reproductive cycle.

**Abstract:**

This study explored the effect of obesity of sows in late pregnancy on metabolic status of perinatal sows and performance, intestinal health, and immune system of offspring piglets. Sixty multiparous Landrance × Large White sows were selected in this study. Sows were divided into two groups according to backfat thickness (normal backfat thickness group, =17 mm; excessive backfat thickness group, ≥21 mm) at days 109 of gestation. The excessive backfat thickness of sows during late pregnancy decreased the total number and litter weight of piglets born alive. Compared with normal backfat thickness sows, the excessive backfat thickness sows had increased levels of plasma glucose, IL-6, and TNF-α and homeostasis model assessment insulin resistance values. The excessive backfat thickness also reduced total superoxide dismutase but increased thiobarbituric acid reactive substances in plasma of perinatal sows. Additionally, the fecal levels of TNF-α were increased but those of IL-10 were decreased in piglets from excessive backfat thickness sow. These findings indicate that the obesity of sows during late pregnancy aggravates the metabolic disorder of perinatal sows, reduces the number of piglets born alive, and adversely affects the intestinal health of sows and their offspring piglets.

## 1. Introduction

In modern large-scale pig farms, the assessment and control of body condition in sows during pregnancy is the key means to optimize their reproductive performance [1]. The backfat thickness is a common indicator of sow body condition [2]. Increasing evidence shows that excessive backfat of sows during late gestation is closely related to reproductive disorders, including less litter size, lower litter weight gain, and more proportion of intrauterine growth restriction [3,4,5]. The latest research indicates that when Landrance × Large White sows have a backfat ≥ 21 mm at days 109 of gestation, the higher m6A modification shown in genes related to placental development may limit development of placental vasculature, thereby increasing the incidence of low birth weight in piglets [6]. Therefore, maintaining backfat during late gestation in proper range is the basic guarantee for maximizing reproductive performance of sows.

Over the course of a normal gestation and lactation, the female body exhibits remarkable metabolic and immunological alterations [7,8]. These changes are described as metabolic syndrome, including insulin resistance and increased levels of systemic pro-inflammatory cytokines in later pregnancy [9,10]. Recently, studies in pregnant women have shown that overweight or obesity during pregnancy may exacerbate the maternal metabolic syndrome [11,12,13]. Furthermore, maternal obesity during pregnancy also increases the risk of obesity and insulin resistance in offspring during childhood or adulthood [11,14]. The increased systemic and intestinal local inflammation and impaired immune development were also observed in offspring of obese pregnant women during pregnancy [15,16]. However, studies on the effects of obesity during late pregnancy on maternal and offspring are mostly reported in women and rodents, and its effects on metabolic status of sows and immune system development of piglets are not clear.

Recently, our study suggested that the perinatal sow’s body also undergoes a systemic low-grade inflammation and increment in insulin resistance [17]. Moreover, the increased intestinal permeability during perinatal period is one of the key mechanisms leading to metabolic disorders in sows [17]. Therefore, based on the above analysis, we hypothesized that excessive backfat thickness of sows at late pregnancy might increase intestinal permeability and metabolic syndrome of perinatal sows and impair intestinal health and immune system development of piglets. The aim of this study was to investigate the effects of excessive backfat thickness of sows at late pregnancy on metabolic status of perinatal sows and performance, intestinal health, and immune system of offspring piglets.

## 2. Materials and Methods

The experimental protocol had been approved by the Institutional Animal Care and Use Committee of Huazhong Agricultural University (permit number: HZAUSW-2018-136).

### 2.1. Animals, Diets, and Experimental Design

The database used in this study was obtained from the research farm of Guangxi Yangxiang Co., Ltd., Guangxi Province, China. The study involved 60 multiparous Landrance × Large White sows with an average parity of 3.93. Sows were divided into two treatment groups according to backfat thickness (normal backfat thickness group, =17 mm; excessive backfat thickness group, ≥21 mm) at 109 d of gestation. In both backfat groups were the similar distribution of parities. Throughout the pregnancy period, all sows were fed the same amount of feed. In detail, the whole pregnancy period was divided into four feeding stages, namely, d 0 to d 30 of pregnancy, d 31 to d 80 of pregnancy, d 81 to d 95 of pregnancy, and d 96 of pregnancy to parturition; correspondingly, the feeding amount was 2.0 kg/d, 2.4 kg/d, 2.6 kg/d, and 3.0 kg/d, respectively. Sows were fed twice per day at 07:00 and 14:00 h. All sows were allowed to consume the same lactation diets ad libitum. The detailed ingredients and nutrient contents of experimental diets are shown in Supplementary Table S1. During pregnancy, sows were housed separately in pregnancy stalls (2.2 × 0.7 × 1.1 m) with 1.2–1.6 m of solid concrete in front and 0.6 to 1.0 m of slotted concrete in the back. On day 109 of pregnancy, sows were washed and then moved from the gestation stalls to the farrowing rooms. Sows were individually housed in farrowing crates (2.2 × 1.7 m) with slatted floors and heat pads for piglets. The backfat thickness of sows were later recorded. During lactation, sows were fed a corn-soybean meal-based lactating diet (10.36 MJ/kg of metabolizable energy, 18.92% of crude protein, and 1.03% lysine). The lactation diet was supplied two times a day at 07:00 and 16:00 to ensure sows’ ad libitum access to feed. The farrowing room temperature was maintained at approximately 20 °C to 22 °C by a water-cooling ventilation system (Big Dutchman, Inc., Vechta-Calveslage, Germany). The sows had ad libitum access to water throughout the experimental period.

At parturition, the number of live-born piglets was recorded. Each piglet was individually weighed whin 12 h after birth. The litter size was then was adjusted to 11–12 in the same backfat treatment group. Piglets were given iron supplementation and cut off their teeth and tails within the first three days life. During lactation, piglets were not fed with creep feed and therefore depended on sow’s milk as their sole source of nutrients. The piglets could not obtain probiotics and antibiotics.

### 2.2. Data Measurements and Collection

Data measurements and collection were conducted by the research team with the help of farm workers. The thickness of backfat was measured by using ultrasound (PIGLOG105, SFAK-Technology, A Mode Scanner, SFK Technology A/S Helver, Denmark) on the last rib (P2; 6.5 cm from the midline of the last rib) and operated by the same employee throughout the experiment according to the method of Sulabo et al. [18]. Body weight of piglets were individually recorded on day of delivery, after cross-fostering and 7, 14, and 21 days of lactation to calculate litter weight and daily gain (ADG). The statistical unit is the litter.

### 2.3. Sample Collection

On day 109 of pregnancy and day 3 of lactation before feeding (0700), 10 sows in each group were randomly selected to collect fasting blood samples from ear veins and mounted in a heparinized centrifuge tube (5 mL). The fasting blood samples were centrifuged for 10 min at 3000× *g* at 4 °C. Plasma samples were then obtained from the supernatant and stored at −80 °C for further analysis. Fresh fecal samples were individually collected from the above sows into sterile 20 mL centrifuge tubes (without any treatment) on day 109 of pregnancy and day 3 of lactation and then stored at −80 °C until analysis. About 4 h after the initiation of farrowing, colostrum samples (20 mL) were collected from the 3 to 5 pairs of mammary glands of the above sows. Milk samples (20 mL) were also taken from the 3 to 5 pairs of mammary glands of sows on day 3 of lactation after an intramuscular injection of 5 IU oxytocin behind an ear. The colostrum and milk samples were immediately frozen at −20 °C until analysis. On day 14 of lactation, one piglet with a body weight close to the average weight of the litter was selected to collect fecal and blood samples. Blood samples were obtained from piglets by vena jugularis with a minimum amount of stress into heparinized tubes (5 mL). Plasma samples were then obtained by centrifuging the blood samples and stored at −80 °C until analysis. Fresh fecal samples were individually collected using sterile 10 mL centrifuge tubes (without any treatment) from the piglets and then stored at −80 °C until analysis.

### 2.4. Chemical Composition of Colostrum and Milk Analysis

Before analysis, colostrum and milk were delipidated by centrifugation at 3000× *g* at 4 °C for 20 min. The chemical composition of colostrum and milk was determined with a near infrared reflectance spectroscopy method by Milk-ScanTM Mars (Foss Electric, Hillerod, Denmark). Th concentrations of IL-6, IL-10, IgA, and IgM in colostrum or milk were determined using porcine commercial assay kits (Bio-Swamp Life Science, Wuhan, China) and their corresponding instructions.

### 2.5. Metabolic and Immune Biomarkers Analysis

In this study, several biomarkers in fecal and plasma related to insulin sensitivity, systemic inflammation, oxidative stress, intestinal permeability, local intestinal inflammation, and immune system of piglets were measured. These biomarkers were as follows: (1) Insulin sensitivity indicators included blood glucose and insulin levels before feeding and HOMA-IR values; (2) systemic inflammation indicators included plasma IL-6, TNF-α, and IL-10 levels; (3) systemic oxidative stress indicators included plasma thiobarbituric acid reactive substances (TBARS), glutathione peroxidase (GPx), and total superoxide dismutase (T-SOD) concentrations; (4) intestinal local inflammation index included fecal lipocalin-2 and fecal inflammatory cytokines (TNF-α, IL-6, and IL-10); (5) intestinal permeability index included plasma zonulin; (6) indicator of immune system development in piglets, fecal β-defensin-2 and secretory IgA (sIgA), and plasma IgM level.

Fecal homogenates were obtained by dissolving feces in PBS (10% wt:vol) and then stored at −80 °C for further analysis. The fecal or plasma concentrations of zonulin, lipocalin-2, β-defensin-2, IL-6, IL-10, TNF-α, sIgA, and IgM were detected using porcine commercial assay kits (Bio-Swamp Life Science, Wuhan, China) and their corresponding instructions. Plasma glucose, insulin, TBARS, glutathione peroxidase (GSH-Px), and T-SOD levels were determined using commercial kits purchased from Nanjing Jiancheng Bioengineering Institute (Nanjing, Jiangsu, China) according to the manufacturer’s instructions. The formula of HOMA-IR values was as follows: HOMA-IR = [(fasting insulin value, mIU/L) × (fasting glucose value, mmol/L)]/22.5. Each sample was determined in duplicate.

### 2.6. Statistical Analysis

Statistical analyses were conducted using the Student *t*-test procedure of SAS 9.4 (SAS Inst. Inc., Cary, NC, USA). The normal distribution of data was verified by a Kolmogorov–Smirnov test. Data were expressed as means ± SEM. A *p* < 0.05 was considered to indicate significance for all analyses.

## 3. Results

### 3.1. Growth Performance of Piglets

Descriptive statistics for backfat thickness and parity of sows and growth performance of suckling piglets during lactation are summarized in Table 1. The total number of piglets born alive and the litter weight of piglets born alive were lower (*p* < 0.05) in the excessive backfat thickness group (≥21 mm) than those in the normal backfat thickness group (=17 mm). The ADG during week 1 was higher (*p* < 0.05) in piglets of excessive backfat thickness group compared with piglets in normal backfat thickness group. However, there was no difference (*p* > 0.05) in ADG during week 2, week 3, or the whole lactation period between the two treatments. Additionally, no difference in the average weight of suckling piglets was observed (*p* > 0.05).

### 3.2. Metabolic Syndrome in Perinatal Sows

**Figure 1:**
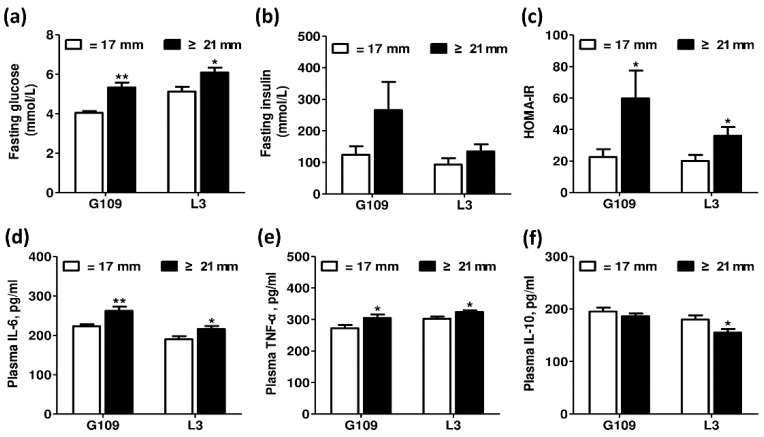
Effect of excessive backfat thickness of sows during late pregnancy on metabolic syndrome in perinatal sows ^1,2^. (**a**) Fasting glucose; (**b**) Fasting insulin; (**c**) HOMA-IR; (d) Plasma IL-6; (**e**) Plasma TNF-α; (**f**) Plasma IL-10. ^1^ Data are expressed as mean ± SEM (*n* = 10). Significance is considered at *p* < 0.05. * effect of treatment (*p* < 0.05), ** effect of treatment (*p* < 0.01); ^2^ G109 = day 109 of pregnancy; L3 = day 3 of lactation; HOMA-IR = homeostasis model assessment of insulin resistance; IL-6 = interleukin 6; TNF-α = tumor necrosis factor α; IL-10 = interleukin 10.

To assess effect of excessive backfat thickness of sows during late pregnancy on metabolic syndrome in perinatal sows, we examined the biomarkers related to insulin sensitivity and systemic inflammation. The results presented in Figure 1 showed that the fasting plasma glucose levels and HOMA-IR values were remarkably increased (*p* < 0.05) in perinatal sows from excessive backfat thickness group when compared with those from normal backfat thickness group. Notably, in excessive backfat thickness sows, the plasma concentrations of pro-inflammatory cytokines IL-6 and TNF-α were increased (*p* < 0.05), while the plasma levels of the anti-inflammatory cytokine IL-10 were decreased (*p* < 0.05).

### 3.3. Oxidative Stress Status of Perinatal Sows

**Figure 2:**
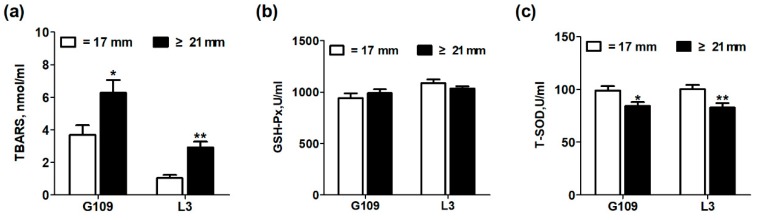
Effect of excessive backfat thickness of sows during late pregnancy on plasma thiobarbituric acid reactive substances (TBARS) (**a**)**,** glutathione peroxidase (GSH-Px) (**b**), and total superoxide dismutase (T-SOD) (**c**) activities in sows ^1,2^. ^1^ Data are expressed as mean ± SEM (*n* =10). Significance is considered at *p* < 0.05. * effect of treatment (*p* < 0.05), ** effect of treatment (*p* < 0.01); ^2^ G109 = day 109 of pregnancy; L3 = day 3 of lactation; TBARS = thiobarbituric acid reactive substances; GSH-Px = glutathione peroxidase; T-SOD = total superoxide disumutase.

The effects of excessive backfat thickness of sows during late pregnancy on oxidative stress status in perinatal sows are shown in Figure 2. Compared with perinatal sows in normal backfat thickness group, the plasma TBARS levels of excessive backfat thickness sows increased significantly (*p* < 0.05), but the plasma T-SOD concentrations decreased significantly (*p* < 0.05). No difference in the plasma GSH-Px levels of perinatal sows was observed (*p* > 0.05).

### 3.4. Intestinal Inflammation and Intestinal Permeability of Perinatal Sows

**Figure 3:**
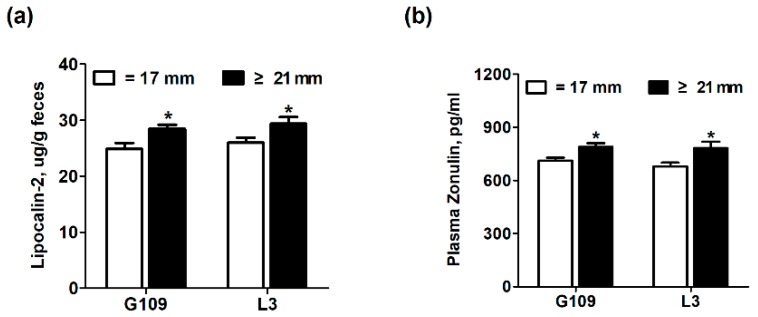
Effect of excessive backfat thickness of sows during late pregnancy on fecal lipocalin-2 (**a**) and plasma zonulin (**b**) activities in sows ^1,2^. ^1^ Data are expressed as mean ± SEM (*n* = 10). Significance is considered at *p* < 0.05. * effect of treatment (*p* < 0.05); ^2^ G109 = day 109 of pregnancy; L3 = day 3 of lactation.

Figure 3 shows the effect of excessive backfat thickness of sows during late pregnancy on intestinal inflammation and intestinal permeability biomarkers in perinatal sows. The zonulin, a protein that participates in tight junctions between cells of the wall in the digestive tract, has been shown to reflect intestinal permeability [19]. At the same time, lipocalin-2, originally appreciated as being highly abundant in neutrophils, is a commonly used sensitive indicator of intestinal inflammation in feces [17,20]. The results showed that the levels of plasma zonulin and fecal lipocalin-2 were remarkably increased (*p* < 0.05) in perinatal sows from excessive backfat thickness group when compared with those from normal backfat thickness group.

### 3.5. Chemical Composition and Immune Factors of Colostrum and Milk in Sows

The effect of excessive backfat thickness of sows in late pregnancy on chemical composition and immune factors of colostrum and milk in sows are shown in Table 2. There was no significant difference (*p* > 0.05) on the chemical composition of colostrum and milk between the two treatments. However, the excessive backfat thickness sows had lower (*p* < 0.05) concentrations of IL-10 in milk than the normal backfat thickness sows. No difference on the levels of IgA, IgM, and IL-6 in colostrum or milk was observed (*p* > 0.05).

### 3.6. Intestinal Inflammation and Intestinal Permeability in Suckling Piglets

**Figure 4:**
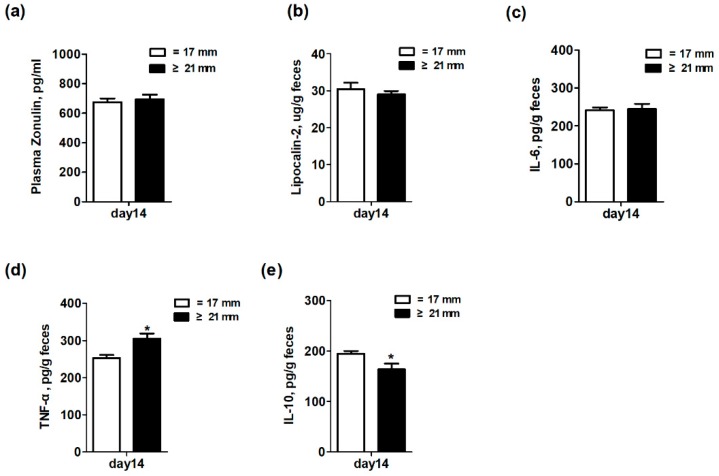
Effect of excessive backfat thickness of sows during late pregnancy on plasma zonulin (**a**) and fecal lipocalin-2 (**b**), IL-6 (**c**), TNF-α (**d**), and IL-10 (**e**) activities in suckling piglets ^1,2^. ^1^ Data are expressed as mean ± SEM (*n* = 10). Significance is considered at *p* < 0.05. * effect of treatment (*p* < 0.05); ^2^ IL-6 = interleukin 6; TNF-α = tumor necrosis factor α; IL-10 = interleukin 10.

The effect of excessive backfat thickness of sows during late pregnancy on intestinal inflammation and intestinal permeability biomarkers of two-week old offspring piglets are displayed in Figure 4. The results showed that the fecal levels of TNF-α were significantly increased (*p* < 0.05) but the fecal levels of IL-10 were remarkably decreased (*p* < 0.05) in piglets from excessive backfat thickness sow when compared with those from normal backfat thickness sow. There was no difference (*p* > 0.05) in levels of plasma zonulin and fecal lipocalin-2 and IL-6 of piglets between the two treatment groups.

### 3.7. Biomarkers of Immune System Development in Suckling Piglets

**Figure 5:**
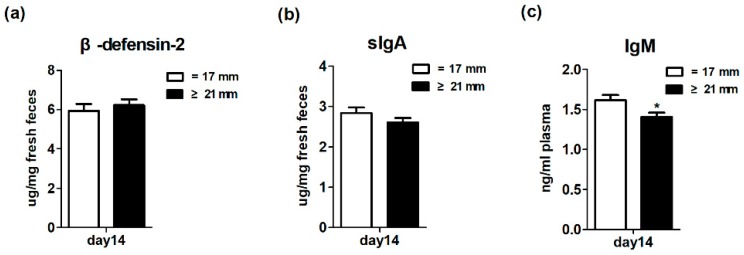
Effect of excessive backfat thickness of sows during late pregnancy on fecal β-defensin-2 (**a**), secretory immunoglobulin A (sIgA) (**b**), and plasma immunoglobulin M (IgM) (**c**) activities in suckling piglets ^1,2^. ^1^ Data are expressed as mean ± SEM (*n* = 10). Significance is considered at *p* < 0.05. * effect of treatment (*p* < 0.05); ^2^ sIgA, secretory immunoglobulin A; IgM, immunoglobulin M.

Figure 5 shows the effects of excessive backfat thickness of sows during late pregnancy on immune system development of two-week old suckling piglets. Three biomarkers related to immune system development were determined. Specifically, fecal β-defensin-2 and sIgA and plasma IgM levels were used to reflect the innate, adaptive, and systemic immune system, respectively [21,22]. The results showed that there was no significant difference (*p* > 0.05) in the fecal levels of β-defensin-2 and sIgA between the two treatments. However, the piglets from excessive backfat thickness sow had lower (*p* < 0.05) concentrations of plasma IgM than piglets from normal backfat thickness sow. This section may be divided by subheadings. It should provide a concise and precise description of the experimental results, their interpretation as well as the experimental conclusions that can be drawn.

## 4. Discussion

In the pig industry, maintaining the optimal body condition of sows is important for achieving the best reproductive efficiency [1]. The backfat thickness has been proven to be an objective, convenient, and precise indicator to reflect the body condition of sows [23,24]. In the current study, our results showed that the excessive backfat thickness (≥21 mm) of Landrance × Large White sows at day 109 of gestation decreased the total number and litter weight of piglets born alive. Similar to the results of this study, Zhou et al. [5] observed that increased backfat thickness of Large White sows at day 109 of gestation had a convex quadratic relationship with litter weight of piglets born alive. Torres-Rovira et al. [25] showed that the proportion of low birth weight in excessive backfat thickness sows was more than 20%. Increasing the rate of low birth weight would reduce the litter weight of piglets at birth. Additionally, Catalano et al. [26] reported that obesity in pregnant women during later pregnancy also increased the incidence of stillbirth and neonatal death. Some studies have shown that the placental dysfunction may be a determinant of maternal obesity during pregnancy leading to abnormal fetal development [5,27,28]. In our previous study, we found that when sows had a backfat ≥ 21 mm at days 109 of gestation, the higher m^6^A modification shown in genes associated with placental development limits the development of placental vasculature, thereby increasing the incidence of low birth weight in piglets [6]. Taken together, our results support the necessity of body condition assessment in sows during pregnancy for optimizing reproductive performance.

In this present study, it was found that the excessive backfat thickness in sows during late pregnancy exacerbated the metabolic disorders in sows, including insulin resistance, systemic inflammation, and oxidative stress in sows during perinatal period. Consistent with this study, Nicholas et al. [11] reported that maternal obesity during pregnancy aggravated their own insulin resistance. Pendeloski et al. [13] also indicated that serum CRP levels, a biomarker for systemic inflammation, increased significantly in obese individuals during pregnancy. In addition, Malti et al. [29] also found that obese pregnant women had increased plasma TBARS levels and significantly decreased systemic T-SOD activity. In this current study, the plasma zonulin and fecal lipocalin-2 levels in perinatal sows with excessive backfat thickness were remarkably increased. Previous studies have suggested that the impaired intestinal mucosal integrity may enhance concentrations of zonulin in blood [30]. Moreover, Lipocalin-2, abundantly enriched in neutrophils, has been reported to be elevated in gut inflammation [20]. This finding suggests that the intestinal permeability and intestinal inflammation were remarkably increased in excessive backfat thickness sows. Our previous studies have confirmed that increased intestinal permeability due to gut microbiota disorders is the potential mechanism for perinatal insulin resistance and systemic inflammation in sows [17]. Thus, these results suggested that obesity of sows at late pregnancy may also contribute to the dysbiosis of gut microbiota in sows, which has been confirmed in obese pregnant women [31,32].

The effect of maternal obesity on intestinal barrier function and immune system of suckling piglets has not been reported before. In this study, we found that excessive backfat thickness in sows during late pregnancy exacerbated intestinal inflammation (fecal levels of TNF-α were increased but IL-10 were decreased) in offspring piglets and reduced the circulatory immune defense capacity of piglets, which was consistent with reports in humans and rodents [14,16,33]. Wilson et al. [16] showed that obesity-induced metabolic disorders during pregnancy not only caused the occurrence of systemic inflammation in the offspring, but also hindered the maturation of the immune system. This study observed a significant decrease in IL-10 levels in normal milk on the third day of the excessive backfat thickness sows. Therefore, changing the transmission of immune factors in milk to offspring may be one of the important ways, but the specific mechanism needs further research.

## 5. Conclusions

The results of this study suggested that excessive backfat thickness in sows during late pregnancy exacerbates the metabolic disorder of sows during perinatal period, reduces the number and litter weight of the piglets born alive, and affects the intestinal health of piglets. Considering the significant effects of body condition during pregnancy on sow metabolism and piglet performance and intestinal health, attention should be paid to the monitoring and control of sow body condition in pig production.

## Figures and Tables

**Table 1:** Effect of excessive backfat thickness of sows in late pregnancy on growth performance of piglets ^1,2^.

Item ^2^	Backfat Thickness at d109 of Gestation		SEM	*p*-Value
	=17 mm	≥21 mm		
Number of sows	30	30		
Backfat thickness at d109 of gestation	17.00	21.80	0.33	<0.01
Parity	3.83	4.03	0.14	0.49
Litter size, NO./litter				
Born alive	14.27	12.60 *	0.41	0.04
After cross-foster	11.97	11.83	0.22	0.76
Litter weight, kg				
At Birth	20.80	18.58 *	0.57	0.05
After cross-foster	17.84	17.81	0.56	0.98
At weaning	64.84	64.33	1.68	0.88
Piglet weight average, kg				
After cross-foster	1.50	1.53	0.01	0.21
At day 7	2.45	2.54	0.02	0.06
At day 14	4.18	4.27	0.04	0.21
At day 21	5.96	6.02	0.05	0.67
ADG of piglet, g/d				
Week 1	134.96	146.34 *	2.30	0.01
Week 2	247.22	247.23	2.94	0.99
Week 3	250.86	255.10	3.78	0.57
Day 1–21	211.96	215.83	2.19	0.38

^1^ All data are shown as mean ± SEM. ^2^ ADG = average daily gain; Wk = Week. * Significance is considered at *p* < 0.05. * effect of treatment (*p* < 0.05).

**Table 2:** Effect of excessive backfat thickness of sows in late pregnancy on chemical composition and immune factors of colostrum and milk in sows ^1,2^.

Items	Backfat Thickness at D109 of Gestation		SEM	*p*-Value
	=17 mm	≥21 mm		
Colostrum, day 1 of lactation				
Total solids, %	20.13	25.23	1.70	0.14
Solid not fat, %	15.96	19.73	1.26	0.14
Protein, %	11.94	15.68	1.39	0.19
Fat, %	2.68	3.53	0.34	0.23
Lactose, %	2.98	2.70	0.30	0.67
IL-6, pg/mL	202.21	197.93	7.93	0.80
IL-10, pg/mL	167.18	154.52	8.29	0.48
Ig A, ug/mL	462.92	551.73	28.14	0.12
Ig M, ng/mL	1.31	1.47	0.05	0.15
Milk, day 3 of Lactation				
Total solids, %	20.26	19.51	1.13	0.76
Solid not fat, %	12.03	11.03	0.38	0.21
Protein, %	6.63	5.45	0.35	0.09
Fat, %	7.55	7.99	0.90	0.82
Lactose, %	4.70	4.93	0.20	0.57
IL-6, pg/mL	207.19	217.39	6.26	0.45
IL-10, pg/mL	192.26	164.42 *	7.15	0.04

* Significance is considered at *p* < 0.05. * effect of treatment (*p* < 0.05).^1^ All data are shown as mean ± SEM (*n* = 10). ^2^ IL-6 = interleukin 6; IL-10 = interleukin 10; IgA, immunoglobulin A; IgM, immunoglobulin M.

## Supplementary Materials

The following are available online at https://www.mdpi.com/2076-2615/10/1/49/s1, Table S1: Ingredients and nutrient composition of experimental gestation and lactation diets (as-fed basis).

## References

[1] Roongsitthichai A., Tummaruk P. (2014). Importance of Backfat Thickness to Reproductive Performance in Female Pigs. Thai J. Vet. Med..

[2] Houde A.A., Methot S., Murphy B.D., Bordignon V., Palin M.F. (2010). Relationships between backfat thickness and reproductive efficiency of sows: A two-year trial involving two commercial herds fixing backfat thickness at breeding. Can. J. Anim. Sci..

[3] Ratky J., Brussow K.P., Egerszegi I., Torner H., Schneider F., Solti L., Manabe N. (2005). Comparison of follicular and oocyte development and reproductive hormone secretion during the ovulatory period in Hungarian native breed, Mangalica, and Landrace gilts. J. Reprod. Dev..

[4] Kim J.S., Yang X.J., Pangeni D., Baidoo S.K. (2015). Relationship between backfat thickness of sows during late gestation and reproductive efficiency at different parities. Acta Agric. Scand. Sect. A Anim. Sci..

[5] Zhou Y.F., Xu T., Cai A.L., Wu Y.H., Wei H.K., Jiang S.W., Peng J. (2018). Excessive backfat of sows at 109 d of gestation induces lipotoxic placental environment and is associated with declining reproductive performance. J. Anim. Sci..

[6] Song T.X., Lu J.X., Deng Z., Xu T., Yang Y., Wei H.K., Li S.Q., Jiang S.W., Peng J. (2018). Maternal obesity aggravates the abnormality of porcine placenta by increasing N-6-methyladenosine. Int. J. Obes..

[7] Nair R.R., Verma P., Singh K. (2017). Immune-endocrine crosstalk during pregnancy. Gen. Comp. Endocrinol..

[8] Luan H., Meng N., Liu P., Feng Q., Lin S., Fu J., Davidson R., Chen X., Rao W., Chen F. (2014). Pregnancy-induced metabolic phenotype variations in maternal plasma. J. Proteome Res..

[9] Barbour L.A., McCurdy C.E., Hernandez T.L., Kirwan J.P., Catalano P.M., Friedman J.E. (2007). Cellular mechanisms for insulin resistance in normal pregnancy and gestational diabetes. Diabetes Care.

[10] Saltiel A.R., Olefsky J.M. (2017). Inflammatory mechanisms linking obesity and metabolic disease. J. Clin. Investig..

[11] Nicholas L.M., Morrison J.L., Rattanatray L., Zhang S., Ozanne S.E., McMillen I.C. (2016). The early origins of obesity and insulin resistance: Timing, programming and mechanisms. Int. J. Obes..

[12] Hernandez-Trejo M., Montoya-Estrada A., Torres-Ramos Y., Espejel-Nunez A., Guzman-Grenfell A., Morales-Hernandez R., Tolentino-Dolores M., Laresgoiti-Servitje E. (2017). Oxidative stress biomarkers and their relationship with cytokine concentrations in overweight/obese pregnant women and their neonates. BMC Immunol..

[13] Pendeloski K.P.T., Ono E., Torloni M.R., Mattar R., Daher S. (2017). Maternal obesity and inflammatory mediators: A controversial association. Am. J. Reprod. Immunol..

[14] Godfrey K.M., Reynolds R.M., Prescott S.L., Nyirenda M., Jaddoe V.W., Eriksson J.G., Broekman B.F. (2017). Influence of maternal obesity on the long-term health of offspring. Lancet Diabetes Endocrinol..

[15] Xue Y., Wang H., Du M., Zhu M.J. (2014). Maternal obesity induces gut inflammation and impairs gut epithelial barrier function in nonobese diabetic mice. J. Nutr. Biochem..

[16] Wilson R.M., Messaoudi I. (2015). The impact of maternal obesity during pregnancy on offspring immunity. Mol. Cell Endocrinol..

[17] Cheng C., Wei H., Yu H., Xu C., Jiang S., Peng J. (2018). Metabolic Syndrome During Perinatal Period in Sows and the Link With Gut Microbiota and Metabolites. Front. Microbiol..

[18] Sulabo R.C., Jacela J.Y., Tokach M.D., Dritz S.S., Goodband R.D., DeRouchey J.M., Nelssen J.L. (2010). Effects of lactation feed intake and creep feeding on sow and piglet performance. J. Anim. Sci..

[19] Tripathi A., Lammers K.M., Goldblum S., Shea-Donohue T., Netzel-Arnett S., Buzza M.S., Antalis T.M., Vogel S.N., Zhao A.P., Yang S.Q. (2009). Identification of human zonulin, a physiological modulator of tight junctions, as prehaptoglobin-2. Proc. Natl. Acad. Sci. USA.

[20] Chassaing B., Srinivasan G., Delgado M.A., Young A.N., Gewirtz A.T., Vijay-Kumar M. (2012). Fecal Lipocalin 2, a Sensitive and Broadly Dynamic Non-Invasive Biomarker for Intestinal Inflammation. PLoS ONE.

[21] Bonder M.J., Tigchelaar E.F., Cai X.H., Trynka G., Cenit M.C., Hrdlickova B., Zhong H.Z., Vatanen T., Gevers D., Wijmenga C. (2016). The influence of a short-term gluten-free diet on the human gut microbiome. Genome Med..

[22] Planer J.D., Peng Y.Q., Kau A.L., Blanton L.V., Ndao I.M., Tarr P.I., Warner B.B., Gordon J.I. (2016). Development of the gut microbiota and mucosal IgA responses in twins and gnotobiotic mice. Nature.

[23] Charette R., BigrasPoulin M., Martineau G.P. (1996). Body condition evaluation in sows. Livest. Prod. Sci..

[24] Maes D.G.D., Janssens G.P.J., Delputte P., Lammertyn A., de Kruif A. (2004). Back fat measurements in sows from three commercial pig herds: Relationship with reproductive efficiency and correlation with visual body condition scores. Livest. Prod. Sci..

[25] Torres-Rovira L., Tarrade A., Astiz S., Mourier E., Perez-Solana M., de la Cruz P., Gomez-Fidalgo E., Sanchez-Sanchez R., Chavatte-Palmer P., Gonzalez-Bulnes A. (2013). Sex and Breed-Dependent Organ Development and Metabolic Responses in Foetuses from Lean and Obese/Leptin Resistant Swine. PLoS ONE.

[26] Catalano P.M., Ehrenberg H.M. (2006). The short- and long-term implications of maternal obesity on the mother and her offspring. BJOG Int. J. Obs. Gynaecol..

[27] Jarvie E., Hauguel-de-Mouzon S., Nelson S.M., Sattar N., Catalano P.M., Freeman D.J. (2010). Lipotoxicity in obese pregnancy and its potential role in adverse pregnancy outcome and obesity in the offspring. Clin. Sci..

[28] Howell K.R., Powell T.L. (2017). Effects of maternal obesity on placental function and fetal development. Reproduction.

[29] Malti N., Merzouk H., Merzouk S.A., Loukidi B., Karaouzene N., Malti A., Narce M. (2014). Oxidative stress and maternal obesity: Feto-placental unit interaction. Placenta.

[30] Fasano A. (2011). Zonulin and its regulation of intestinal barrier function: The biological door to inflammation, autoimmunity, and cancer. Physiol. Rev..

[31] Santacruz A., Collado M.C., Garcia-Valdes L., Segura M.T., Martin-Lagos J.A., Anjos T., Marti-Romero M., Lopez R.M., Florido J., Campoy C. (2010). Gut microbiota composition is associated with body weight, weight gain and biochemical parameters in pregnant women. Br. J. Nutr..

[32] Mokkala K., Roytio H., Munukka E., Pietila S., Ekblad U., Ronnemaa T., Eerola E., Laiho A., Laitinen K. (2016). Gut Microbiota Richness and Composition and Dietary Intake of Overweight Pregnant Women Are Related to Serum Zonulin Concentration, a Marker for Intestinal Permeability. J. Nutr..

[33] Broadney M.M., Chahal N., Michels K.A., McLain A.C., Ghassabian A., Lawrence D.A., Yeung E.H. (2017). Impact of parental obesity on neonatal markers of inflammation and immune response. Int. J. Obes..

