# Supplementary material for: Obesity of Sows at Late Pregnancy Aggravates Metabolic Disorder of Perinatal Sows and Affects Performance and Intestinal Health of Piglets
Source: Animals (Basel). 2019 Dec 25;10(1):49. doi: 10.3390/ani10010049 (PMC7023453; doi:10.3390/ani10010049)
Supplement: Supplementary file 1 [file animals-10-00049-s001.pdf]

Table S1. Ingredients and nutrient composition of experimental gestation and lactation diets (as-fed basis).

Ingredients	Gestation diet	Lactation diet
Ingredient, %		
Corn	54.80	54.40
Soybean meal, 43%CP	11.50	26.00
Wheat bran	0.00	11.00
Soybean hull	14.00	0.00
Rice bran meal	16.00	0.00
Calcium carbonate	1.20	1.51
Dicalcium phosphate	1.00	1.23
Salt	0.40	0.26
Mildewcide[1]	0.10	0.10
Choline chloride	0.00	1.00
Premix[2]	1.00	1.50
Nutrient composition		
Net energy, MJ/kg[3]	9.53	10.36
Crude protein, %	13.96	18.92
ISF, %[4]	26.95	14.45
SF, %[5]	2.85	1.76
NDF, %[6]	15.70	11.18
ADF, %[7]	5.47	4.26
Lys, %[3]	0.59	1.03
Ca, %[3]	0.78	1.06
Available P, %[3]	0.40	0.45

[1]Mildewcide: ammonium propionate.

[2]Provided per kg of diet: Cu, 30 mg; Fe, 160 mg; Zn, 160 mg; Mn, 55 mg; I, 0.5 mg; Se, 0.5 mg; Co, 0.8 mg; Cr, 0.2 mg; Vitamin A, 14000 IU; Vitamin D_3, 2900 IU; Vitamin E, 120 mg; Vitamin K_3, 6 mg; Vitamin B_1, 2.4 mg; Vitamin B_2, 8.5 mg; Vitamin B_6, 4.5 mg; Vitamin B_{12}, 0.03 mg; Vitamin H, 0.55 mg; Pantothenic acid, 30 mg; Folic acid, 5 mg; Nicotinamide, 50 mg.

[3]Calculated chemical concentrations using values for feed ingredients from the NRC (2012).

[4]insoluble fiber.

[5]solubel fiber.

[6]neutral detergent fiber.

[7]acid detergent fiber.
